# Reproductive and fetal toxicity studies of histamine H3 receptor antagonist DL76 used in mice to prevent maximal electroshock-induced seizure

**DOI:** 10.3389/fphar.2024.1364353

**Published:** 2024-06-05

**Authors:** Salim M. Bastaki, Yousef M. Abdulrazzaq, Marwan Abdelrahim Zidan, Mohamed Shafiullah, Saif Ghdayer Alaryani, Fatima Awad Alnuaimi, Ernest Adeghate, Sahar Mohsin, Amal Akour, Agata Siwek, Dorota Łażewska, Katarzyna Kieć-Kononowicz, Bassem Sadek

**Affiliations:** ^1^ Department of Pharmacology and Therapeutics, College of Medicine and Health Science, United Arab Emirates University, Al Ain, United Arab Emirates; ^2^ Zayed Center for Health Sciences, United Arab Emirates University, Al Ain, United Arab Emirates; ^3^ Department of Paediatrics and Neonatology, College of Medicine and Health Science, United Arab Emirates University, Al Ain, United Arab Emirates; ^4^ Department of Education, Dubai Health Authority, Dubai, United Arab Emirates; ^5^ Department of Anatomy, College of Medicine and Health Sciences, United Arab Emirates University, Al Ain, United Arab Emirates; ^6^ Department of Pharmacobiology, Faculty of Pharmacy, Jagiellonian University Medical College in Kraków, Kraków, Poland; ^7^ Department of Technology and Biotechnology of Drugs, Faculty of Pharmacy, Jagiellonian University Medical College in Kraków, Kraków, Poland

**Keywords:** histamine H3 receptors, antagonist DL76, maximal electroshock, seizures, anticonvulsant, malformation, gestation, mice

## Abstract

**Introduction:** Brain histamine is considered an endogenous anticonvulsant and histamine H1 receptor. H1R antagonists have, in earlier studies, been found to induce convulsions. Moreover, research during the last two decades has provided more information concerning the anticonvulsant activities of histamine H3R (H3R) antagonists investigated in a variety of animal epilepsy models.

**Methods:** Therefore, the *in vivo* anticonvulsant effect of the H3R antagonist DL76, with proven high *in vitro* affinity, *in vitro* selectivity profile, and high *in vivo* antagonist potency in mice against maximal electroshock (MES)-induced seizures in mice, was assessed. Valproic acid (VPA) was used as a reference antiepileptic drug (AED). In addition, DL76 was tested for its reproductive and fetal toxicity in the same animal species.

**Results and discussion:** Our observations showed that acute systemic administration (intraperitoneal; i.p.) of DL76 (7.5 mg/kg, 15 mg/kg, 30 mg/kg, and 60 mg/kg, i.p.) provided significant and dose-dependent protection against MES-induced seizures in female and male mice. Moreover, the DL76-provided protective effects were comparable to those offered by the VPA and were reversed when animals were co-administered the CNS-penetrant selective H3R agonist *R*-(α)-methylhistamine (RAM, 10 mg/kg, i.p.). Furthermore, the administration of single (7.5 mg/kg, 15 mg/kg, 30 mg/kg, or 60 mg/kg, i.p.) or multiple doses (3 × 15 mg/kg, i.p.) of H3R antagonist DL76 on gestation days (GD) 8 or 13 failed to affect the maternal body weight of mice when compared with the control mice group. No significant alterations were detected in the average number of implantations and resorptions between the control and DL76-treated groups at the early stages of gestation and the organogenesis period. In addition, no significant differences in the occurrence of skeletal abnormalities, urogenital abnormalities, exencephaly, exomphalos, facial clefts, and caudal malformations were observed. The only significant abnormalities witnessed in the treated groups of mice were in the length of long bones and body length. In conclusion, the novel H3R antagonist DL76 protected test animals against MES-induced seizures and had a low incidence of reproductive and fetal malformation with decreased long bone lengths *in vivo*, signifying the potential therapeutic value of H3R antagonist DL76 for future preclinical as well as clinical development for use in the management of epilepsy.

## 1 Introduction

Epilepsy is characterized by seizures that are unpredictable in frequency and is the second most common neurological disorder that affects people of all ages, with onset most often occurring in childhood and older adulthood (Mauritz et al., 2022; Yang et al., 2022; Asadi-Pooya et al., 2023). Epilepsy leads to abnormal behavioral paradigms, necessitating a lifelong treatment with effective antiepileptic drugs (AEDs) (Brigo et al., 2013; Mauritz et al., 2022). The anticipated prevalence rate of epilepsy in the United States and other regions is high. Approximately 1.5 million women with epilepsy give birth to 27,000 infants yearly, with an estimated prevalence of epilepsy in pregnant women of approximately 0.5% (Tomson and Battino, 2007; Thomas et al., 2012; Sadek et al., 2014a; Sadek et al., 2015; Bastaki et al., 2018; Tomson et al., 2018). Clinical observations revealed that approximately 60%–70% of patients diagnosed with epilepsy respond to available treatments with AEDs. However, resistance to monotherapy is not uncommon, and a combination of several AEDs is often inevitable with the possibility of drug–drug interactions (Brigo et al., 2013; Sadek et al., 2014a). Following chronic clinical use of several AEDs, the incidence of major malformations in infants of epileptic parents was found to be twice that of non-epileptic parents (Sadek et al., 2015; Bastaki et al., 2018). In addition, gestational epilepsy is a concern for women with a pre-existing seizure and can lead to a 17% rise in seizure episodes (Tomson et al., 2018). Accordingly, hormonal fluctuations and changes in the pharmacokinetics of AEDs play a crucial role (Thomas et al., 2012). Therapeutic drug monitoring and maintaining strict adherence to AEDs have improved therapeutic management (Pennell et al., 2020), yet the threat of complications for both mother and child persists. Epilepsy can increase maternal mortality fivefold, and *in utero* exposure to AEDs, notably valproic acid (VPA), increases the risk of congenital defects, which is also related to drug type and dosage and is worsened by specific drug combinations (Viale et al., 2015).

The involvement of the brain histaminergic neurotransmitter system in seizure control is well-established, as several previous observations in preclinical experiments demonstrated that brain histamine is capable of modulating seizure trends in both electrically and chemically induced seizure models in animals (Kamei et al., 1993a; Kamei et al., 1993b; Chen et al., 2002; Zhang et al., 2003; Sadek et al., 2014b). Accordingly, the precursor of brain histamine, namely, the amino acid L-histidine, was reported to decrease chemically induced seizure in experimental animals by stimulating the brain histaminergic neurotransmission and increasing the seizure threshold by interactions of brain histamine with postsynaptically located histamine H1 receptors (H1Rs) in the brain (Chen et al., 2002). Moreover, the involvement of central H1Rs in the development of seizures was confirmed by preclinical studies that showed an increased tendency of seizures in mice lacking H1Rs or histidine decarboxylase enzyme responsible for the biosynthesis of histamine in the brain (Jang et al., 2010; Miyata et al., 2011). Clinically, it has been found that the use of high doses of various centrally acting H1R antagonists belonging to old-generation antihistamines, such as diphenhydramine, as antiallergic drugs occasionally increased the risk of convulsions in healthy young children, and this risk was especially observed among long-term users (Ago et al., 2006; Jang et al., 2010; Miyata et al., 2011). Histamine interacts with four G-protein-coupled H1-4R subtypes. H3R, first described in 1983, was found to regulate histamine biosynthesis and release, acting as presynaptic auto-receptors (Arrang et al., 1983; 1987; Brown et al., 2001). Accordingly, H3R antagonists or histamine *N*-methyl transferase (HNMT) inhibitors (e.g., methoprene) were found to increase brain histamine levels and decrease seizures in epileptic patients through interactions with H1Rs (Witkin and Nelson, 2004; Vohora et al., 2010). Hence, the potential of H3R antagonists as future AEDs has begun to be increasingly considered, as mounting evidence from both acute and chronic experimental seizure models demonstrated the anticonvulsant efficacy of numerous imidazole- and non-imidazole-based H3R antagonists, such as thioperamide, 2–18, DL77, E159, and E169 (Brown et al., 2001; Vohora et al., 2010; Witkin and Nelson, 2004; Sadek et al., 2015; Bastaki et al., 2018).

**FIGURE 1:**
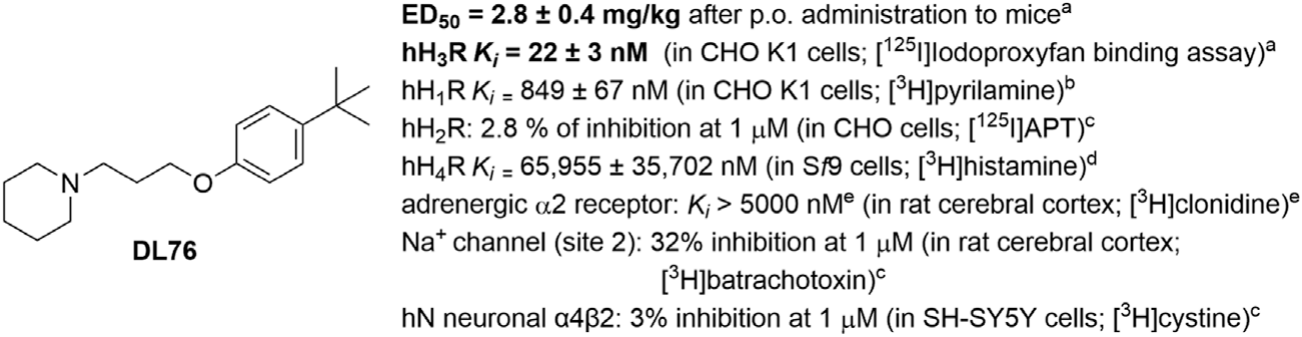
Chemical structure, *in vitro* affinities, and *in vivo* H3R antagonist potency of DL76. ^a^Data from Lazewska et al. (2006). ^b^Measured according to a previous protocol (Lazewska et al., 2017). ^c^Radioligand binding studies were performed commercially in Eurofins Cerep Laboratories (Celle-Lévescault, France). ^D^Data from Lazewska et al. (2020) and Canale et al. (2021). ^e^Measured according to a previous protocol (Canale et al., 2021).

Based on the afore-mentioned preclinical and clinical findings, and in search of potent and selective histamine H3R antagonists with anticonvulsant properties, our research group succeeded in developing the histamine H3R antagonist DL76 [1-(3-(4-tert-butyl-phenoxy) propyl) piperidine] (Figure 1), a non-imidazole-based H3R ligand, which proved to be a highly potent H3R antagonist (human H3R *K*_i_ = 22 nM) with good selectivity profile vs. other histamine receptors and high oral potency in mice with an ED_50_ value of 2.8 ± 0.4 mg/kg (Lazewska et al., 2006; Lazewska et al., 2017; Lazewska et al., 2020; Canale et al., 2021) (Figure 1). Moreover, previous research works clearly showed antiparkinsonian effects of H3R antagonist DL76 without inducing *in vitro* toxicity when tested in several cell lines (Canale et al., 2021). In a previous preclinical pharmacokinetic evaluation study in experimental animals, concentrations of DL76 in the cortex, hippocampus, and striatum were found to be significantly higher (44%) than in the whole brain tissues. Following administration of the doses, C_0_ values increased from 564 ng/mL (3 mg/kg) to 653 ng/mL (6 mg/kg), while AUC0→∞ was found to be doubled when the tested dose increased from 3 mg/kg to 6 mg/kg (298 ng◦h/mL vs. 685 ng◦h/mL) (Szafarz et al., 2015). Therefore, the H3R antagonist DL76, which has a high *in vitro* H3R antagonist affinity, an excellent *in vitro* selectivity profile, a high *in vivo* H3R antagonist potency, and pharmacokinetics and tissue distribution profiles, was selected for further investigations of its anticonvulsant efficacy in MES-induced seizure models in male and female adult mice. Possible reproductive toxicities in the same animal species were assessed following systemic administration of H3R antagonist DL76 in a wide range of doses.

## 2 Materials and methods

### 2.1 Animals

Experiments utilized 8–10-week-old C57BL/6J mice (30–35 g) from The Jackson Laboratory, referred to as C57 mice, of both sexes. Mice were group-housed in standard Plexiglas cages with *ad libitum* access to food and water in a 12-h light/dark cycle (lights on at 6:00 a.m.). Procedures were conducted between 9:00 a.m. and 12:00 p.m., following guidelines of the European Communities Council Directive (86/609/EEC) and approved by the Institutional Animal Ethics Committee for epilepsy (ERA-2017-5535) and teratogenic studies (A14-14) at the College of Medicine and Health Sciences/United Arab Emirates University.

### 2.2 Drugs

The reference AED VPA and the CNS-penetrant H3R agonist (*R*)-α-methylhistamine (RAM, 10 mg/kg, i.p.) were obtained from Sigma-Aldrich (St Louis, Missouri, USA). The test compound DL76 was synthesized by the Department of Technology and Biotechnology of Drugs (Kraków, Poland) according to previously described synthetic protocols (Lazewska et al., 2006). The test compound DL76, the reference drug VPA (300 mg/kg), and the CNS-penetrant H3R agonist RAM (10 mg/kg) were dissolved in isotonic saline and administered intraperitoneally (i.p.) at a volume of 1 mL/kg for all *in vivo* studies. DL76 was used in a dose range of 7.5 mg/kg, 15 mg/kg, 30 mg/kg, and 60 mg/kg. All doses of used drugs were expressed in terms of the free bases. Eight mice were used to assess the anticonvulsant activity of each compound.

### 2.3 Maximal electroshock (MES)-induced seizure

As previously described and with a slight modification in the intensity applied, seizures were elicited in mice with a 50-Hz alternating current of 120 mA intensity (Kaminski et al., 2015; Song et al., 2020; Xiao et al., 2021; Hua et al., 2023). The current was applied through ear electrodes for 0.2 s. Protection against the spread of MES-induced convulsion was defined as the abolition of the tonic hind limb extension (THLE) component of the seizure (Kaminski et al., 2015; Song et al., 2020; Xiao et al., 2021; Hua et al., 2023). The animals were divided into the following experimental groups, with eight mice in each: the control group injected with saline, the positive control group injected with 300 mg/kg of VPA (the minimal dose of VPA that protected animals against the spread of MES-induced convulsions without mortality in mice) (Sadek et al., 2015; Bastaki et al., 2018), and four groups that were administered the test compound DL76 at doses of 7.5 mg/kg, 15 mg/kg, 30 mg/kg, or 60 mg/kg. All drugs were administered 30–45 min before the MES challenge. In a further group of eight mice, the most promising and protective dose of DL76 (60 mg/kg, i.p.) was co-injected with RAM (10 mg/kg, i.p.), 30 min apart and 15 min prior to the MES test. Protection against the spread of MES-induced convulsion was defined as the abolition of the THLE component of the convulsion (Sadek et al., 2015; Alachkar et al., 2018; Bastaki et al., 2018).

### 2.4 Reproductive studies

Reproductive studies were conducted in adult female mice, averaging 30 gm in weight and 6 weeks in age (Padmanabhan et al., 2006; Bastaki et al., 2018). Following successful mating, indicated by vaginal plug observation, the plug-positive day was considered gestation day 0 (GD-0). DL76 was administered at various doses (7.5 mg/kg, 15 mg/kg, 30 mg/kg, and 60 mg/kg) through single i.p. injections and three 15 mg/kg injections in 1 day to mouse groups on GD-8 and GD-13 (Tables 1, 2). The control group was injected with normal saline. Appropriate formation of different organs occurs in specific periods of the development of the mouse. These two periods were selected because GD-8 is known to be a critical period for the induction of neural tube defects, craniofacial malformations, and the development of other organs, whereas on GD-13, the retina and other parts are being formed, and most other organs have been formed. The number of implantations, fetal deaths, and resorptions was recorded. Fetal and placental weights were documented separately. After blotting dry, the fetuses were weighed and fixed in 95% ethanol. The reproductive toxicological effects of DL76 were assessed by observing gross and visceral malformations, following Sterz and Lehmann (1985), Abdulrazzaq et al. (1997), Padmanabhan et al. (2003), and Padmanabhan et al. (2006).

**TABLE 1:** Morphological effect of systemic treatment of H3R antagonist DL-76 on gestation day (GD-8) in C57/B6 mouse embryos.

		DL76 (mg/kg) on GD-8				
	Control	7.5	15	30	60	3 × 15-dose
Total no. of embryos	38	44	38	51	46	47
Mandible hypoplasia	2 (5.3)	3 (6.8)	3 (7.9)	3 (5.9)	4 (8.7)	3 (6.4)
Maxilla hypoplasia	1 (2.6)	3 (6.8)	1 (2.6)	2 (3.9)*	2 (4.3)	2 (4.3)
Eye open	1 (2.6)	2 (4.5)	3 (7.9)	2 (3.9)	1 (2.2)	4 (8.5)
Microtia/low set microtia	1 (2.6)	2 (4.5)	1 (2.6)	2 (3.9)	2 (4.3)	2 (4.3)
Exomphalos	1 (2.6)	0	1 (2.6)	1 (2.0)	0	0
Left/right kidney hypo/descended	1 (2.6)	1 (2.3)	2 (5.3)	2 ((3.9)	2 (4.3)	2 (4.3)
Undescended testis	0	2 (4.5)	0	0	0	1 (2.1)
Number of male embryos	21 (55.3)	25 (56.8)	18 (47.4)	24 (47.1)	25 (54.3)	23 (48.9)
Number of female embryos	17 (44.7)	19 (43.2)	20 (52.6)	27 (52.9)	21 (45.7)	24 (51.1)
Kinky tail	0	0	0	1 (2.0)	0	0

^*^ *p* < 0.05 compared to the values of the corresponding controls; Parentheses contain percentages. Control mice were administered with saline.

**TABLE 2:** Morphological effect of systemic treatment of drug DL-76 on gestation day (GD-13) in C57/B6 mouse embryos.

		DL76 (mg/kg) on GD-13				
	Control	7.5	15	30	60	3 × 15-dose
Total no. of embryos	47	50	40	43	32	48
Mandible hypoplasia	3 (6.4)	4 (8.0)	4 (10.0)	3 (7.0)	2 (6.3)	3 (6.3)
Maxilla hypoplasia	2 (4.3)	4 (8.0)*	3 (7.5)	2 (4.7)	2 (6.3)	3 (6.3)
Eye open	0	3 (6.0)	2 (5.0)	0	2 (6.3)	6 (12.5)
Microtia/low set microtia	2 (4.3)	2 (4.0)	2 (5.0)	1 (2.3)	2 (6.3)	3 (6.3)
Exomphalos	0	0	0	1 (2.3)	0	1 (2.1)
Left/right kidney hypo/descended	1 (2.1)	2 (4.0)	1 (2.5)	1 (2.3)	2 (6.3)	2 (4.2)
Undescended testis	0	1 (2.0)	1 (2.5)	0	2 (6.3)	0
Number of male embryos	22 (46.8)	23 (46.0)	22 (55.0)	19 (44.2)	15 (46.9)	24 (50.0)
Number of female embryos	25 (53.2)	27 (54.0)	18 (45.0)	24 (55.8)	17 (53.1)	24 (50.0)
Kinky tail	0	0	0	0	0	1 (2.1)

*p < 0.05 compared to the values of the corresponding controls; Parentheses contain percentages. Control mice were administered with saline.

#### 2.4.1 Method of whole embryo observation

Deformities of the embryos were identified according to a modified method of Sterz and Lehmann (1985), Abdulrazzaq et al. (1997), and Bass et al. (2020). Accordingly, the embryos were removed from the 95% ethanol, and the lower part of the abdomen was cut using a razor blade. All organs were checked for abnormalities with a stereo dissecting microscope, and finally, the organs were removed by using forceps.

#### 2.4.2 Double staining methods

After isolating fetuses from viscera and skin, they underwent a fat-removal process by immersion in acetone for 1–3 days. The resulting transparent specimens underwent a sequence of glycerin solutions (50% and 80%) before being finally stored in 100% glycerin for observation of malformations using a stereo dissecting microscope (McLeod, 1980; Sterz and Lehmann, 1985; Abdulrazzaq et al., 1997; Bass et al., 2020). Following this, specimens were processed and stained with Alizarin Red-S and Alcian blue (McLeod, 1980; Sterz and Lehmann, 1985; Abdulrazzaq et al., 1997; Bass et al., 2020) to identify bone and cartilage deformities.

### 2.5 Statistics

Statistical analyses employed SPSS 26.0 (IBM Middle East, Dubai, UAE). Data were presented as means ± SEM. Convulsion effects, measured by THLE in seconds, and reproductive toxicities were assessed using one-way ANOVA with treatment (vehicle or test compound) and dose (test compound) as between-subjects factors, followed by least significant difference (LSD) *post hoc* analysis. Fetal abnormality data were presented as counts and percentages. Fisher’s exact test compared the percentages of abnormalities between the control group (saline) and the different dose groups. All tests were two-tailed, and significance was set at *p* < 0.05.

## 3 Results

### 3.1 Protective effects of H3R antagonist DL76 against MES-induced convulsions in male adult mice

**FIGURE 2:**
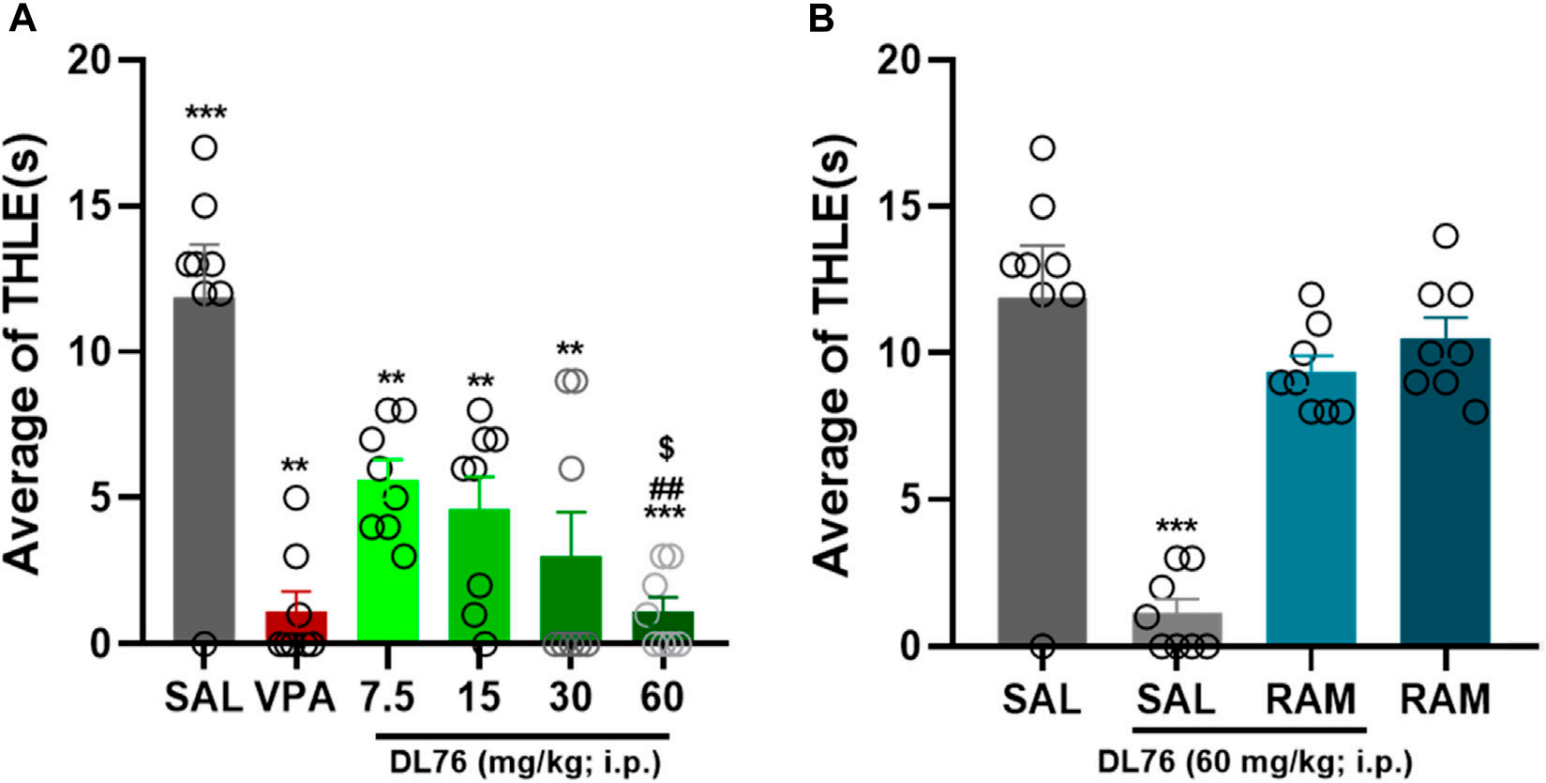
Anticonvulsant effect of acute systemic administration of H3R antagonist DL76 on MES-induced seizure in male adult mice. **(A)** Anticonvulsant effects of valproic acid (VPA, 300 mg/kg, i.p.) and test compound DL76 (7.5 mg/kg, 15 mg/kg, 30 mg/kg, and 60 mg/kg, i.p.) on the duration of tonic hind limb extension (THLE) induced in an MES model in male adult mice. **(B)** Effect of CNS-penetrant H3R agonist RAM (10 mg/kg, i.p.) pretreatment on the protection by H3R antagonist DL76 (60 mg/kg, i.p.) on MES-induced convulsions in male adult mice. Each value represents mean ± SEM (n = 8). ^***^ *p* < 0.001 *versus* saline- and DL76 (7.5 mg/kg)-treated groups. ^##^ *p* < 0.001 *versus* DL76 (7.5 mg/kg)- and DL76 (15 mg/kg)-treated groups. ^$^ *p* < 0.05 *versus* DL76 (30 mg/kg)-treated group.

The acute systemic administration of a dose range of DL76 (7.5 mg/kg, 15 mg/kg, 30 mg/kg, and 60 mg/kg, i.p.) exhibited a protective effect on MES-induced convulsions [F (7,56) = 16.54; *p* < 0.0001] (Figure 2). The results show that there are substantial protective effects of all doses of DL76 (i.e., 7.5 mg/kg, 15 mg/kg, 30 mg/kg, and 60 mg/kg when compared with saline (all *p* values <0.0001) (Figure 2). Among the doses tested, the 60 mg/kg dose of DL76 showed the most promising protection in an MES model in adult male mice when compared with the saline-, DL76 (7.5 mg/kg)-, DL76 (15 mg/kg)-, and DL76 (30 mg/kg)-treated groups [mean difference of THLE 6.25 s, 7.25 s, 8.88 s, and 10.75 s; respectively, all *p* < 0.0001] (Figure 2). Notably, the protection provided by H3R antagonist DL76 at the higher dose (60 mg/kg, i.p.) was comparable to that provided by the reference drug VPA (*p* = 1.000) (Figure 2) and was reversed following co-injection with the CNS-penetrant histamine H3R agonist RAM (10 mg/kg, i.p.) 15 min before the MES challenge, with a mean difference of 2.5 s (*p*-value = 0.094), for the comparison of saline vs. DL76+RAM (Figure 2). Interestingly, RAM (10 mg/kg, i.p.), when injected alone, did not affect MES-induced convulsions (*p* = 0.602 saline + saline vs. DL76 (60 mg/kg) + RAM) (Figure 2). Figure 2 represents the significant differences between all doses of DL76 and compared to VPA (300 mg/kg), RAM, and DL76 60 mg/kg + RAM observed in adult male mice.

### 3.2 Protective effects of H3R antagonist DL76 against the MES-induced convulsion model in female adult mice

**FIGURE 3:**
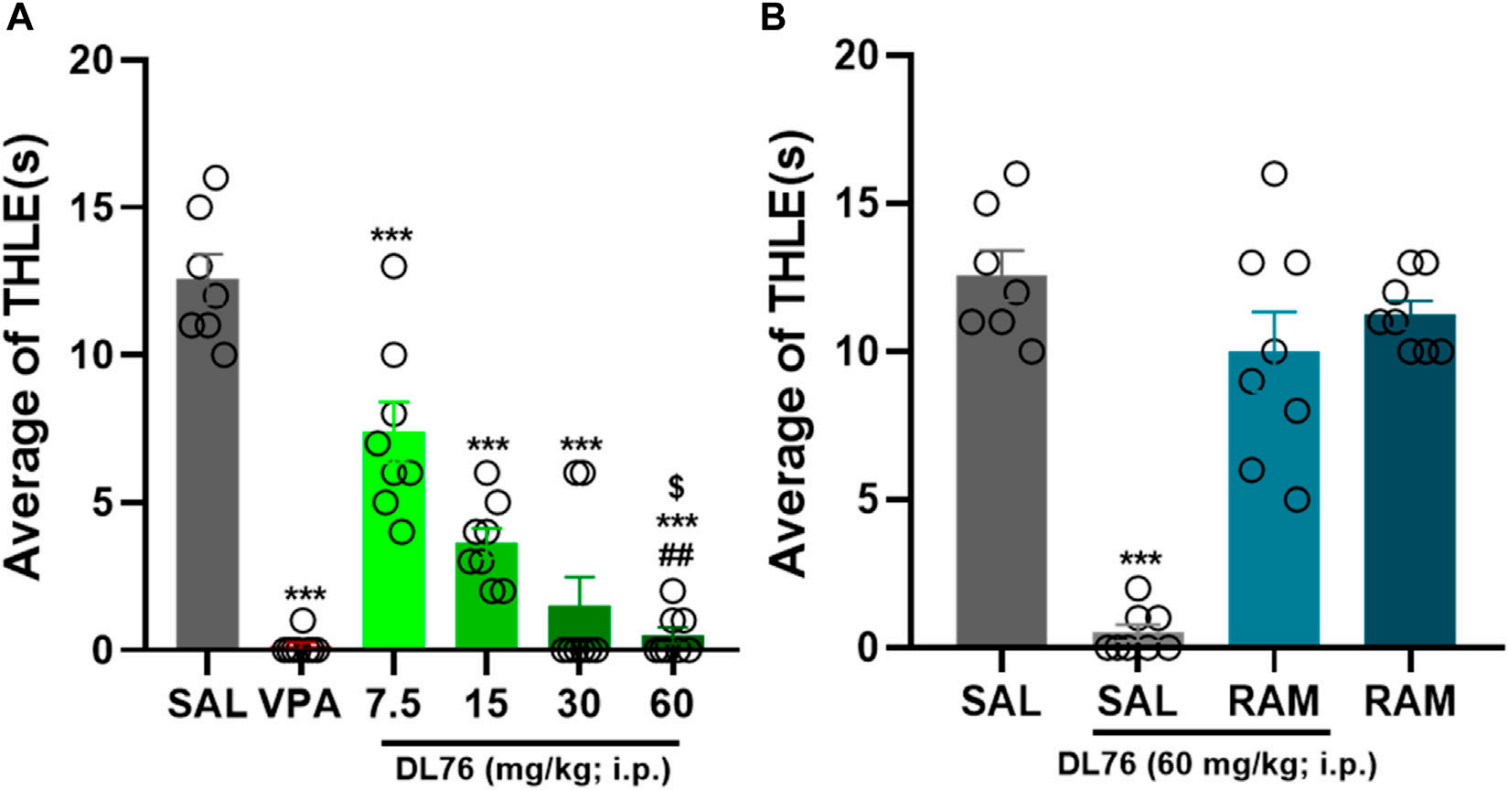
Anticonvulsant effect of acute systemic administration of H3R antagonist DL76 on MES-induced seizure in female adult mice. **(A)** Anticonvulsant effects of valproic acid (VPA, 300 mg/kg, i.p.) and test compound DL76 (7.5 mg/kg, 15 mg/kg, 30 mg/kg, and 60 mg/kg, i.p.) on the duration of tonic hind limb extension (THLE) induced in an MES model in female adult mice. **(B)** Effect of CNS-penetrant H3R agonist RAM (10 mg/kg, i.p.) pretreatment on the protection by H3R antagonist DL76 (60 mg/kg, i.p.) on MES-induced convulsions in female adult mice. Each value represents mean ± SEM (n = 8). ^***^ *p* < 0.001 *versus* saline- and DL76 (7.5 mg/kg)-treated groups. ^##^ *p* < 0.001 *versus* DL76 (7.5 mg/kg)- and DL76 (15 mg/kg)-treated groups. ^$^ *p* < 0.05 *versus* DL76 (30 mg/kg)-treated group.

The results showed that VPA (300 mg/kg) and DL76 (7.5–60 mg/kg, i.p.) significantly protected animals against MES-induced convulsions [F (7,56) = 51.78; *p* < 0.0001] (Figure 3). In addition, the results showed that DL76 at a dose of 60 mg/kg significantly provided the highest protection in an MES model in female adult mice when compared with the saline-, DL76 (7.5 mg/kg)-, DL76 (15 mg/kg)-, and DL76 (30 mg/kg)-treated groups [mean difference in THLE of 12.13 s, 10.63 s, and 3.13 s; respectively (*p* < 0.05)] (Figure 3). Similarly, the protection provided by DL76 at the highest dose used in the current study (60 mg/kg, i.p.) was comparable to that provided by the reference drug VPA (300 mg/kg) (*p* = 0.72) and was abrogated following acute systemic co-administration of RAM (10 mg/kg, i.p.) 15 min before the MES challenge [mean difference = 1.33; *p* = 0.17, for the comparison of saline + saline vs. DL76 + RAM] (Figure 3). Data showed that RAM alone in female adult mice generated results similar to those witnessed in male adult mice (10 mg/kg, i.p.; *p* = 0.91 saline-saline vs. saline-RAM) (Figure 3). Interestingly, no significant differences were observed for the protective effects provided in male and female mice after acute systemic administration of VPA (300 mg/kg, i. p.) or H3R antagonist DL76 at doses of 7.5 mg/kg, 15 mg/kg, 30 mg/kg, and 60 mg/kg, i.p. [F (1,14) = 2.15; *p* = 0.164, for the comparison of saline + VPA in male mice vs. saline + VPA in female mice], [F (1,14) = 0.156; *p* = 0.70, for the comparison of saline + DL76 (7.5 mg/kg) in male mice vs. saline + DL76 (7.5 mg/kg) in female mice], [F (1,14) = 0.17; *p* = 0.685, for the comparison of saline + DL76 (15 mg/kg) in male mice vs. saline + DL76 (15 mg/kg) in female mice], [F (1,14) = 2.31; *p* = 0.151, for the of comparison saline + DL76 (30 mg/kg) in male mice vs. saline + DL76 (30 mg/kg) in female mice], and [F (1,14) = 0.76; *p* = 0.398, for the comparison of saline + DL76 (60 mg/kg) in male mice vs. saline + DL76 (60 mg/kg) in female mice], respectively.

### 3.3 Results of reproductive studies

There was no sign of maternal toxicity in any of the mice from all groups in both the GD-8 and GD-13 treated animals. The number of fetuses in each litter was not significantly different between all study groups, nor was the number of live fetuses. The resorption rate was not significantly different between all the groups. The mean fetal weight in each litter was not significantly different between all treatment groups and the controls. The mean placental weights were also not significantly different between the treated groups and the controls. The incidence of gross morphological anomalies in the treated fetuses of the single- and multiple-dose groups given i.p. was not significantly different from that in the control group. There was no significant difference in the incidence of exencephaly and craniofacial malformations, such as mandibular and maxillary hypoplasia, exomphalos, low set microtia, exophthalmia, eye remaining open, posterior bilateral palate, posterior unilateral palate, hydronephrosis, descended kidney, undescended testis, or kinky tail, between any of the groups studied (Tables 1, 3).

**TABLE 3:** Effect of systemic treatment of H3R antagonist DL-76 on gestation day (GD-8) in C57/B6 mouse embryos.

			DL76 (mg/kg)				
	Non-Treat. Con	Control	7.5	15	30	60	3 × 15-dose
Number of litters	7	6	7	6	7	7	7
Fetuses/litter	9.143 ± 1.574	7.333 ± 2.066	7.714 ± 1.890	7.167 ± 2.317	8.429 ± 0.787	7.286 ± 1.380	8.143 ± 0.690
Live fetus/litter	8.143 (89.06)	6.333 (86.36)	6.289 (81.48)	6.338 (88.37)	7.286 (86.44)	6.571 (90.20)	6.714 (82.46)
Resorption/litter	1.000 (10.94)	1.000 (13.64)	1.429 (18.51)	0.833 (11.62)	1.143 (13.56)	0.714 (9.80)	1.429 (17.54)
Fetal weight/							
Litter (g) (Mean ± SD)	1.009 ± 0.012	1.050 ± .0.071	1.143 ± 0.107	0.993 ± 0.106	1.004 ± 0.047	1.048 ± .0156	1.037 ± 0.101
IUGR −1SD	1/57 (1.75)	1/38 (2.63)	0	0	1/51 (1.96)	0	3/47 (6.38)
IUGR −2SD	0	1/38 (2.63)	0	2/38 (5.26)	0	2/51 (4.35)	0
Placental weight/							
Litter (g) (mean ± SD)	0.104 ± .011	0.104 ± 0.014	0.109 ± 0.11	0.105 ± 0.009	0.097 ± 0.019	0.101 ± 0.010	0.092 ± 0.007
IUGR −1SD	1/57 (1.75)	1/38 (2.63)	0	0	2/51 (3.92)	1/51 (2.17)	3/47 (4.26)
IUGR −2SD	0	0	0	0	1/51 (1.96)	0	0

Note: Non-Treat. Con = Non-treated control; Parentheses contain percentages. Control mice were administered with saline.

### 3.4 Results of skeletal malformations

**FIGURE 4:**
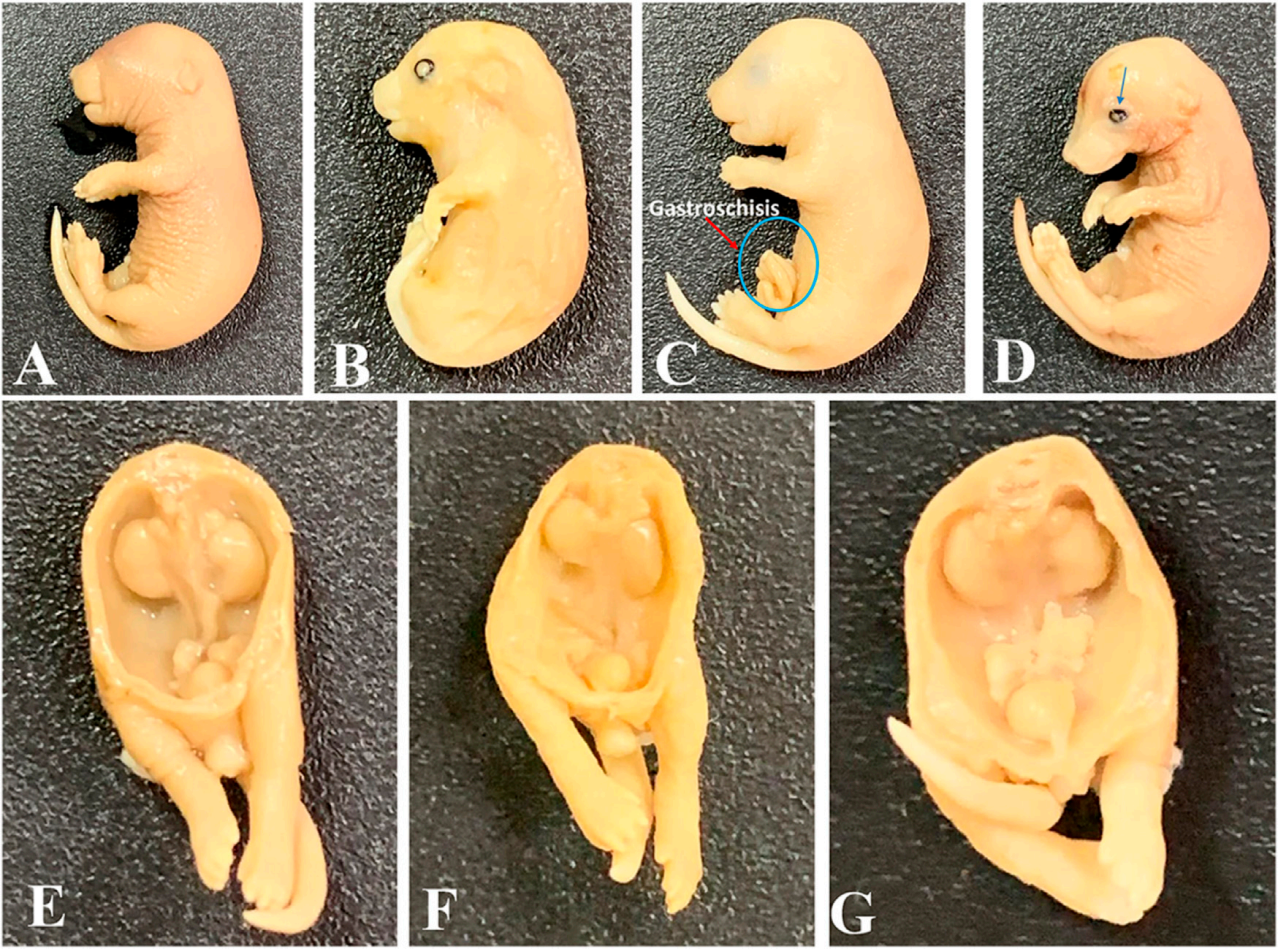
Some abnormalities detected in tested mice embryos at gestational day 18. **(A)** Normal embryo; **(B)** mandibular and maxillary hypoplasia with an open eye; **(C)** embryo with gastroschisis; **(D)** intrauterine growth restriction (IUGR) and open eye; **(E)** normal urogenital system; **(F)** embryo with a hypoplastic right kidney and a descended left kidney; **(G)** embryo with undescended testes. Magnification: ×1.25.

Observations of skeletal defects showed that mean crown-to-rump length decreased significantly in the 7.5 mg/kg-, 30 mg/kg-, and 60 mg/kg-treated groups when compared to control groups. Humerus and ulna lengths were reduced in the 30 mg/kg and 60 mg/kg groups, with only the 30 mg/kg group showing a significant decrease in radius length. Femur length significantly decreased in the 7.5 mg/kg and 60 mg/kg groups; a non-significant decrease (*p* = 0.06) occurred in the 30 mg/kg group. Tibia and fibula lengths significantly decreased in the 7.5 mg/kg, 30 mg/kg, and 60 mg/kg groups when compared to the controls (Tables 1, 2). No significant differences were observed in skull or facial bones (Tables 1, 2). In addition, in the vertebrae, our observed results showed that there were no differences in the number of centra or the number of unossified/hypoplastic bodies in all the DL-76-treated groups. There was no hypoplasia or poor ossification of the caudal vertebrae in any of the groups. The number of coccygeal vertebral bodies was the same in all tested mice groups (Tables 1, 2). The cervical ribs were attached to the seventh cervical vertebra and ended free anteriorly, becoming the first thoracic ribs. The lumbar ribs attached to the first lumbar vertebra were shorter than the last pair of thoracic ribs. The incidence of lumbar ribs in the DL76-treated group was not significantly different when compared to the saline-treated control mice groups. In addition, there was no fusion of ribs, reduction in number and size, or splitting and forking of ribs in either the control or the DL76-treated mice groups. Notably, 13 pairs of thoracic ribs were observed in both control and experimental embryos (Tables 1, 2). Furthermore, the sternum of the control fetuses consisted of seven sternebrae. There was no significant difference in the fifth sternebrium between the DL76-treated and control mice groups with regards to the absence or the occurrence of hypoplasia. We did not observe any malalignment, hemilateral or unilateral agenesis, scrambling, or fusion, or any cases of bifid sternebrae in any of the groups (Tables 1, 2). Figure 4 shows some of the malformations seen in the embryos.

## 4 Discussion

H3R antagonist DL76 showed very promising results in terms of its ability to exert protective effects against MES-induced convulsions in male and female adult mice, especially when mice were pretreated with 60 mg/kg i.p., as compared with the saline-treated groups or other mice groups that received lower doses (Figures 2–4). Therefore, the results demonstrated a dose–response relationship of the protective effects provided by H3R antagonist DL76 against convulsions in adult mice of both sexes (Figure 2, 3). Notably, the DL76 (60 mg/kg)-provided protective effect was similar to that observed for the reference drug VPA (300 mg/kg, i.p.) (Figure 2, 3). The latter observations are in harmony with previous preclinical studies that showed dose-dependent protective effects of a couple of H3R antagonists against MES convulsions induced in several animal models (Sadek et al., 2013; Sadek et al., 2014a; Sadek et al., 2014b; Sadek et al., 2015; Sadek et al., 2016a; Sadek et al., 2016b).

In a very recent study, a group of compounds known as 6-aminoalkoxy-3,4-dihydroquinolin-2(1H)-ones were synthesized to evaluate their potential as H3R antagonists and their ability to prevent seizures (Hua et al., 2023). The synthesized compounds exhibited strong H3R antagonism. Specifically, compounds labeled 2a, 2c, 2h, and 4a demonstrated exceptionally potent H3R antagonistic activities, with half-maximal inhibitory concentrations (IC_50_) of 0.52 μM, 0.47 μM, 0.12 μM, and 0.37 μM, respectively. When tested in the MES model, three compounds—2h, 4a, and 4b—were identified as having antiseizure properties. Molecular docking studies involving compounds 2h, 4a, and a reference compound PIT with the H3R protein were conducted to predict how these molecules might interact with the H3R binding site. The docking results indicated that 2h, 4a, and PIT likely share a similar binding mode to the H3R, providing insights into the molecular basis for their antagonistic effects (Hua et al., 2023).

In addition, several non-imidazole H3R antagonists have been tested and, of these, some, such as E159 and E177, emerged as particularly effective, reducing the duration of THLE in a dose-dependent manner in MES-induced seizures in rats (Sadek et al., 2014b; Alachkar et al., 2018). Previous clinical studies showed that the H3R antagonist PIT exhibited protective effects against the photosensitivity seizure model in adult patients (Lamb, 2020).

The current study showed that the protection observed for H3R antagonist DL76 was almost nullified when animals were co-injected with the CNS-penetrant histamine H3R agonist RAM (10 mg/kg i.p.) (Figure 2B, 3B). However, when injected alone, RAM (10 mg/kg) showed neither a protective nor an epileptogenic activity in mice challenged by the MES-induced convulsion (Figure 2B, 3B). These results suggest that the protective effects of H3R antagonist DL76 in the MES-induced convulsions are mediated, at least in part, through an H3R blockade, which is consistent with the formerly observed protective activities of various H3R antagonists (Brigo et al., 2013; Sadek et al., 2014b; Mauritz et al., 2022). These later effects can be devised from the inhibitory effect of H3Rs on the biosynthesis and release of histamine in the presynaptic histaminergic terminals (Sadek et al., 2014b). Subsequently, blockage of these H3 auto-receptors by selective antagonists, such as our compound H3R antagonist DL76, would result in an enhanced neuronal release of brain histamine, providing the proposed protective effect in the MES-induced convulsion status in mice. Notably, the abrogation effect on numerous previously assessed H3R antagonists brought about either by H3R agonists or by CNS-penetrant H1- or H2R antagonists was described, providing further evidence about the role of the H3R antagonism-released brain histamine (Sadek et al., 2013; Sadek et al., 2014a; Sadek et al., 2014b; Sadek et al., 2015; Sadek et al., 2016a; Sadek et al., 2016b). Interestingly, no significant differences were observed for the reference drug VPA (300 mg/kg, i.p.) and the 30 mg/kg and 60 mg/kg doses of the H3R antagonist DL76 in both sexes of adult mice, but there was a significant difference between the THLE values of VPA and the lower doses of DL76 (i.e., the 7.5 mg/kg, and 15 mg/kg, i.p.). The latter results are discrepant with previous studies in which there was a variation in seizure threshold between both sexes due to differences in levels of steroids and steroidal derivatives, such as 3α-hydroxylated pregnane steroids, which can interact with GABA receptor complex, and therefore, decrease seizure susceptibility in female adult mice (Belelli et al., 1990a; Belelli et al., 1990b; Lan et al., 1990; Maguire et al., 2005). In a further series of experiments, H3R antagonist DL76 was administered at various doses to groups of mice on gestational days 8 and 13 (Tables 1–4). The observed results showed that there was no difference in the abnormalities that occurred in the fetuses. The latter observation is crucial, as all AEDs, including the third-generation agents given to pregnant women, have been shown to have deleterious effects on fetuses in numerous worldwide studies (Abdulrazzaq et al., 1997; Holmes et al., 2001; Padmanabhan et al., 2003; Kaplan, 2004; Padmanabhan et al., 2006).

**TABLE 4:** Effect of systemic treatment of H3R antagonist DL-76 on gestation day (GD-13) in C57/B6 mouse embryos.

	Non-Treat. Con	Saline control	DL76 (mg/kg)				
			7.5	15	30	60	3 × 15-dose
Number of litters	7	7	7	7	7	6	7
Fetuses/litter	9.143 ± 1.574	7.571 ± 0.900	8.000 ± 1.291	7.571 ± 1.272	7.000 ± 1.155	7.000 ± 1.414	8.286 ± 1.704
Live fetus/litter	8.143 (89.06)	6.714 (88.68)	7.143 (76.19)	5.714 (75.47)	6.143 (87.76)	5.333 (76.19)	6.857 (82.76)
Resorption/litter	1.000 (10.94)	0.857 (11.32)	0.857 (10.71)	1.857 (24.53)	.857 (12.24)	1.667 (23.81)	1.429 (17.24)
Fetal weight							
Litter (g) (Mean ± SD)	1.006 ± 0.012	1.040 ± 0.090	1.018 ± 0.103	0.998 ± 0.063	1.069 ± 0.082	1.051 ± 0.122	1.051 ± 0.031
IUGR −1SD	1/57 (1.75)	1/47 (2.13)	1/50 (2.00)	1/40 (2.50)	1/43 (2.33)	0	0
IUGR −2SD	0	1/47 (4.26)	0	0	0	0	0
Placental weight/							
Litter (g) (mean ± SD)	0.104 ± .011	0.101 ± 0.007	0.105 ± 0.011	0.113 ± 0.015	0.103 ± 0.006	0.097 ± 0.010	0.110 ± 0.012
IUGR −1SD	1/57 (1.75)	1/47 (2.13)	1/50 (2.00)	1/40 (2.50)	1/43 (2.33)	1/32 (3.13)	1/48 (2.08)
IUGR −2SD	0	0	0	0	0	1/32 (3.13)	0

Note: Non-treat. Con = Non-treated control; Parentheses contain percentages.

Although some abnormalities were detected in some of the embryos, their numbers were not different than those in the control groups, except for the crown–rump length and lengths of the long bones of the extremities, which seemed to be affected at the higher doses of 30 mg/kg, 60 mg/kg, and 15 mg/kg × 3. No significant signs of maternal toxicity or fetal resorption were seen in the treated groups (Figure 4). The absence of significant abnormalities in the embryos of mice treated with H3R antagonist DL76 in this study is similar to the lack of teratogenic effects of H3R antagonist 2–18 on mice; the only difference is the adverse effects on the bones of the extremities, which seemed to be shorter in the higher dose groups than the controls (Bastaki et al., 2018). Many women abandon AEDs during pregnancy for fear of their effects on the fetus and are, therefore, at great risk of seizure occurrence and morbidity in both the mother and fetus. The H3R antagonist DL76 appears to be relatively safe except for its effects on the length of the long bones of the extremities at high doses. One of the limitations of this study was that it should have compared the antiseizure and anti-toxicity activities of DL76 to a reference H3R antagonist drug. This will be considered in future experiments.

Unfortunately, it is difficult to extrapolate these findings to humans, and therefore, it is difficult to select a dose that would be safe in humans. Considering these findings, it is reasonable to conclude that the safety profile of H3R antagonist DL76 is obviously higher than that of the other AEDs and comes close to the safety profile of other H3R antagonists like 2–18 (Bastaki et al., 2018).

In conclusion, the H3R antagonist DL76 shows the prospect of being a useful protective anticonvulsant drug with potential use during pregnancy, given the fact that AED VPA was described to induce malformations in C57BL/6 mice (Chateauvieux et al., 2010). Nevertheless, further preclinical studies are necessary to determine if these findings can be replicated when administering the H3R antagonist DL76 throughout the entire gestation period, both in the same species and in other animal models.

## Data Availability

The original contributions presented in the study are included in the article/Supplementary Material; further inquiries can be directed to the corresponding authors.
